# Genetic underpinnings of the heterogeneous impact of obesity on lipid levels and cardiovascular disease

**DOI:** 10.1186/s13073-025-01522-9

**Published:** 2025-10-06

**Authors:** Daeeun Kim, Heather M. Highland, Roelof A. J. Smit, Micah R. Hysong, Victoria L. Buchanan, Kristin L. Young, Chi Zhao, Cassandra N. Spracklen, Tuomas O. Kilpeläinen, Boya Guo, Burcu F. Darst, Yanwei Cai, Zhe Wang, Jessica Lundin, Sonja I. Berndt, JoAnn E. Manson, Eirini Marouli, Leslie Lange, Ethan Lange, Myriam Fornage, Christopher R. Gignoux, Christopher A. Haiman, Stephen S. Rich, Steven Buyske, Ruth J. F. Loos, Charles Kooperberg, Ulrike Peters, Christy L. Avery, Penny Gordon-Larsen, Mariaelisa Graff, Laura M. Raffield, Kari E. North

**Affiliations:** 1 Department of Epidemiology, Gillings School of Global Public Health, University of North Carolina at Chapel Hill, Chapel Hill, NC USA; 2 Department of Genetics, School of Medicine, University of North Carolina at Chapel Hill, Chapel Hill, NC USA; 3 Department of Epidemiology, School of Public Health, University of Texas Health Science Center at Houston, Brownsville, TX USA; 4 Charles Bronfman Institute for Personalized Medicine, Icahn School of Medicine at Mount Sinai, New York, NY USA; 5 Novo Nordisk Foundation Center for Basic Metabolic Research, University of Copenhagen, Copenhagen, Denmark; 6 Department of Biostatistics and Epidemiology, University of Massachusetts Amherst, Amherst, MA USA; 7 Division of Public Health Sciences, Fred Hutchinson Cancer Center, Seattle, WA USA; 8 Division of Cancer Epidemiology and Genetics, National Cancer Institute, National Institutes of Health, U.S. Department of Health and Human Services, Bethesda, MD USA; 9 Department of Medicine, Brigham and Women’s Hospital, Harvard Medical School, Boston, MA USA; 10 Barts and The London School of Medicine and Dentistry, William Harvey Research Institute, Queen Mary University of London, London, UK; 11 Division of Biomedical Informatics and Personalized Medicine, Department of Medicine, University of Colorado Anschutz Medical Campus, Aurora, CO USA; 12 Brown Foundation Institute of Molecular Medicine, University of Texas Health Science Center at Houston, Houston, TX USA; 13 Department of Preventive Medicine, University of Southern California, Los Angeles, CA USA; 14 Center for Public Health Genomics, University of Virginia Charlottesville, Charlottesville, VA USA; 15 Department of Genetics, Rutgers University, Piscataway, NJ USA; 16 Department of Nutrition, The University of North Carolina at Chapel Hill, Chapel Hill, NC USA; 17 Border Health Research Center, UTHealth Houston School of Public Health, 780 Ringgold Road, Brownsville, TX 78520‑4979 USA; 18 Department of Internal Medicine, McGovern Medical School, UTHealth Houston School of Public Health, 780 Ringgold Road, Brownsville, TX 78520‑4979 USA

**Keywords:** Heterogeneities in obesity, BMI-lipid pleiotropy, Local genetic correlation, Polygenic risk scores, Mendelian randomization

## Abstract

**Background:**

Obesity is thought to increase cardiovascular disease (CVD) risk partly through dyslipidemia. Yet, obesity’s effects on dyslipidemia are not uniform. Understanding the shared genetic basis between obesity and lipid traits can provide insight into this heterogeneity and its implications for CVD risk.

**Methods:**

We examined local genetic correlations between three lipid measures [high-density lipoprotein cholesterol (HDL), low-density lipoprotein cholesterol (LDL), and triglycerides (TG)] and body mass index (BMI) using genome-wide association study summary statistics from European ancestry UK Biobank participants. We identified genomic loci with opposing genetic effects on obesity and dyslipidemia risk (protective BMI-lipid loci) and those with concordant directions for both obesity and dyslipidemia risk (adverse BMI-lipid loci). Gene-based association analyses were used to prioritize potential causal genes. We then constructed polygenic risk scores for BMI (PRS_BMI_) based on protective and adverse loci and assessed their associations with BMI, lipid levels, CVD, and related traits in the diverse Population Architecture using Genomics and Epidemiology (PAGE) study. PheWAS was performed in the All of Us cohort. Mendelian randomization (MR) was conducted to assess the causal impact of protective/adverse loci on cardiometabolic outcomes. Finally, we investigated the associations with fat distribution traits using MRI-based fat measures in the UK Biobank.

**Results:**

Among 2495 regions, we identified 789 HDL, 26 LDL, and 494 TG loci with significant local genetic correlation with BMI (including overlapping loci). Of these, 3 HDL, 10 LDL, and 8 TG loci showed protective correlations. Gene-based analyses prioritized 18 candidate causal genes. The protective PRS_BMI(+)HDL(+)_ was associated with higher BMI but favorable lipid profiles and reduced CVD risk in PAGE. PheWAS revealed protective associations with hyperlipidemia, atrial fibrillation, and Alzheimer’s disease. MR supported the favorable causal effects of these protective loci on several cardiometabolic outcomes. Notably, protective PRS_BMI(+)TG(−)_ was uniquely associated with decreased visceral-to-abdominal subcutaneous adipose tissue ratio.

**Conclusions:**

Identifying and validating genomic loci with shared genetic signals between BMI and lipid levels further supports the importance of genetics in defining the heterogeneous impact of obesity on dyslipidemia and CVD.

**Supplementary Information:**

The online version contains supplementary material available at 10.1186/s13073-025-01522-9.

## Background

Obesity imposes an enormous global public health burden [1, 2] and increases the burden of cardiovascular diseases (CVD) and many other downstream sequelae [3] through its impact on CVD risk factors (e.g., dyslipidemia, hypertension, and type 2 diabetes) [4–7]. However, major research gaps remain. Few studies have evaluated the often described but poorly understood heterogeneity in CVD outcomes observed among populations with obesity [8]. This heterogeneity may be due to heterogeneous relationships between obesity and CVD risk factors, especially lipid levels [9, 10]. One plausible but largely unexplored source of heterogeneity in the obesity-lipid level relationship is the shared genetic architecture across obesity and lipid traits. Better characterization of the shared genetic underpinnings may help us better understand the heterogeneous impact of obesity on lipid traits and CVD.

Recent studies have suggested that pleiotropic obesity loci, especially those with counterintuitively protective associations with CVD traits, could help explain the observed heterogeneous impact of obesity on CVD [11–17]. For example, two recent studies identified 36 [15] and 62 [11] variants that were linked to both increased adiposity and favorable metabolic profiles, respectively. Several variant-level approaches have been implemented to identify pleiotropic obesity variants [11]; however, no previous studies have used locus-level approaches and local genetic correlation analysis, an emerging genomic analysis tool to explore pleiotropy. In addition, previously identified pleiotropic loci have not been validated in populations with diverse ancestries. As is common with other genetic research, these loci were discovered in European ancestry populations, and it is unknown whether the identified bivariate loci show comparable influences on obesity and cardiometabolic traits in other ancestries.

We therefore aimed to (1) identify genomic regions with significant shared genetic signals between body mass index (BMI) and lipid traits (BMI-lipid bivariate loci) in opposing directions and investigate the potential causal genes underlying counterintuitive pleiotropy between BMI and lipid levels, and (2) examine the potential influence of BMI-lipid bivariate loci on BMI, lipid levels, and downstream CVD and other disease outcomes in diverse populations.

## Methods

### Study cohorts and data sources

We utilized GWAS summary statistics from the UK Biobank (UKBB) for the identification of BMI-lipid bivariate loci. In addition, individual-level data from the Population Architecture using Genetics and Epidemiology (PAGE) study, All of Us (AoU) Research Program, and UKBB were used for testing the associations of the BMI-lipid bivariate loci with relevant phenotypes. Specifically, PAGE data were used to evaluate associations with cardiometabolic traits, AoU data were used to perform a phenome-wide association (PheWAS) analysis, and UKBB data were used to characterize the loci in relation to MRI-derived measures of fat distribution.

#### UKBB

The UKBB is a large-scale prospective study of More than 500,000 individuals Living in the UK. Participants aged 40–69 were recruited from 2006 to 2010, and their phenotypic and genotypic information, including questionnaires, physical and blood measures, genome-wide genotyping data, imaging data, and health outcomes, has been collected [18].

In the current study, we utilized the publicly available Pan-UKBB GWAS summary statistics [19, 20] for BMI and lipid traits in individuals of European ancestry as the discovery sample for identifying BMI-lipid bivariate loci (*N* = 419,163 for BMI, *N* = 367,021 for HDL, *N* = 400,223 for LDL, and *N* = 400,639 for TG). Additional descriptions on the GWAS analysis conducted by the Pan-UKBB team can be found in Additional file 1. We also used MRI-derived regional body fat measures from the UKBB imaging substudy extension [21] to characterize the identified BMI-lipid bivariate loci (maximum available *N* = 22,988).

#### PAGE

The PAGE consortium was launched in 2008 through the NHGRI’s effort to expand the ancestral diversity in genomic studies [22, 23]. PAGE cohort studies include the Atherosclerosis Risk in Communities (ARIC) study, Coronary Artery Risk Development in Young Adults study (CARDIA), Hispanic Community Health Study / Study of Latinos (HCHS/SOL), Women’s Health Initiative (WHI), Multiethnic Cohort Study (MEC), and Icahn School of Medicine at Mount Sinai BioMe biobank. In our study, a total of 83,376 participants with relevant genetic and phenotypic data from these studies were included. Self-identified White (*N* = 25,418), Black (*N* = 25,255), Hispanic/Latino (*N* = 25,814), and East Asian (*N* = 6889) participants were included in the current analysis (Table S1) as the validation population for identified BMI-lipid bivariate loci. These cohort studies are summarized in Additional file 1.

#### AoU

We used the AoU Research Program as the target population for a PheWAS analysis. AoU is a diverse population-based research study that utilizes electronic health records (EHR) from participants across the US. Of the 245,394 participants in the “All of Us Controlled Tier Dataset v7” with whole genome sequencing (WGS) data at the time of this study, 147,327 had demographic data. We excluded those with insufficient EHR data (an EHR length of less than 3 years, with fewer than three independent visit dates), leaving 99,409 participants available for analysis. AoU performed ancestry prediction using gnomAD v3.1 [24] to train a random forest classifier using 1000 Genomes / Human Genome Diversity Project (HGDP) data [25, 26] as a reference. Based on this classification, 61,393 participants were of European ancestry, 20,191 of African ancestry, 15,479 of American ancestry, 1182 of East Asian ancestry, 841 of South Asian ancestry, and 323 of Middle Eastern ancestry. The mean age of the study population was 56.0 (SD: 16.8) years (Table S2).

### Measurement

#### Genetic data

##### UKBB

A total of 488,377 participants from the UKBB were genotyped on the Applied Biosystems UKBB Lung Exome Variant Evaluation (UK BiLEVE) Axiom Array (*N* = 49,950) or the UKB Axiom Array (*N* = 438,427) [27]. Imputation was performed using IMPUTE4 with the Haplotype Reference Consortium, UK10K, and 1000 Genome Phase 3. Detailed methods have been described previously [27].

##### PAGE

Participants were genotyped on the Multi-Ethnic Genotyping Array (MEGA) at the Center for Inherited Disease Research as part of the PAGE study [23, 28]. Additionally, some participants from ARIC, BioMe, CARDIA, MEC, and WHI were genotyped separately on Illumina or Affymetrix arrays by each study or ancillary study. The number of samples included in our analyses by study, self-reported race/ethnicity, and genotyping platform is shown in Table S3. A total of 34,373 samples that were included in the current analysis were Genotyped on the MEGA array, and the remaining 49,003 samples were genotyped on alternative arrays. Table S4 summarizes the genotyping platform, QC criteria, imputation methods, and reference panel that each study and ancillary study implemented.

##### AoU

WGS for all consented participants was performed with NovaSeq 6000. The DRAGEN (Illumina) pipeline (v3.4.12) [29] was utilized to generate the metrics for the data processing steps and to perform genome Mapping, alignment, and variant calling. Over 702 million variants were reported, with an average coverage of ≥ 30× and over 90% of bases at 20× coverage. A consistent sample and data processing protocol was implemented to minimize possible batch effects across centers. Further details on WGS can be found in the AoU Research Program Genomic Research Data Quality Report [30] and other sources [31].

#### Phenotype data

##### UKBB

BMI (weight (kg)/[height (m)]^2^) was calculated from standing height and weight measured at the baseline visit [18]. Participants’ blood samples were also collected at the same visit, and various biomarkers, including three lipid traits (high-density lipoprotein cholesterol (HDL), low-density lipoprotein cholesterol (LDL), and triglycerides (TG)), were measured as previously described [32].

We utilized magnetic resonance imaging (MRI)-derived body fat distribution data to investigate the potential implications of the identified heterogeneous BMI-lipid bivariate loci. As part of the UKBB imaging substudy extension [21], regional fat depositions were quantified using a Siemens MaGNETOM Aera 1.5-T MRI scanner (Siemens Healthineers, Erlangen, Germany) with the dual‐echo Dixon Vibe protocol employed [33, 34]. The sample size and the distribution of variables included in the current analysis are presented in Table S5. The fat distribution variables used in the current study included abdominal visceral adipose tissue volume (VAT), abdominal subcutaneous adipose tissue volume (ASAT), total adipose tissue volume (TAT; defined between the bottom of the thigh muscles and the top of the vertebrae T9), muscle fat infiltration (MFI), and liver fat (assessed using the 10-point symmetric chemical-shift encoded acquisition (10P) method, quantified as the average liver proton density fat fraction (PDFF) across three to nine regions of interest). Additionally, gluteofemoral adipose tissue (GFAT) was derived by subtracting VAT and ASAT from the TAT, as suggested by a previous study [35]. We also computed adipose tissue ratios, including VAT/ASAT, VAT/GFAT, ASAT/GFAT, VAT/TAT, ASAT/TAT, and GFAT/TAT.

##### PAGE

BMI was used as a continuous proxy measure of overall adiposity, and obesity status was defined as BMI ≥ 30 kg/m^2^ for non-East Asians and BMI ≥ 25 kg/m^2^ for East Asians [36, 37]. Blood samples were drawn following an 8-h fast for lipid, glucose, and insulin measures. Three lipid measures (HDL, LDL, and TG) were used as continuous proxy measures of dyslipidemia. HDL and TG levels were directly quantified. Since directly measured LDL was not consistently available across all PAGE participants, LDL levels were instead computed using the Friedewald Eq. [38], excluding individuals whose TG levels were > 400 mg/dL, as in previous literature [39]. We determined participants’ diabetes status if they met any of the following American Diabetes Association criteria [40]: diabetes medication, self-reported diagnosis, fasting glucose ≥ 7 mmol/L or HbA1c ≥ 48 mmol/mol, or random glucose > 11.11 mmol/L, and aged ≥ 25 years at the time of diagnosis (to avoid potential misclassification between T1D and T2D). Blood pressure was measured with a standardized protocol. We classified participants as hypertensive if they met any of the following criteria: (1) systolic blood pressure (SBP) ≥ 140 mmHg, (2) diastolic blood pressure (DBP) ≥ 90 mmHg, (3) self-reported use of any antihypertensive medication, or (4) ICD-9 codes 401.x or ICD-10 codes I10.x—I15.x [23]. Additionally, a subset of PAGE cohorts collected CVD data, including prevalence, incidence, or related deaths. Detailed descriptions of phenotypic measures for each cohort in PAGE are provided in Additional file 1.

### Statistical analyses

#### Global SNP-based heritability and genetic correlations

Prior to performing local genetic correlation analyses, we estimated the global single nucleotide polymorphism (SNP)-based heritability of BMI and the three lipid traits, as well as the genetic correlation between three BMI-lipid pairs (BMI-HDL, BMI-LDL, and BMI-TG) in the UKBB by performing linkage disequilibrium (LD) score regression [41] based on GWAS summary statistics from UKBB.

#### Bivariate loci identification

**Fig. 1:**
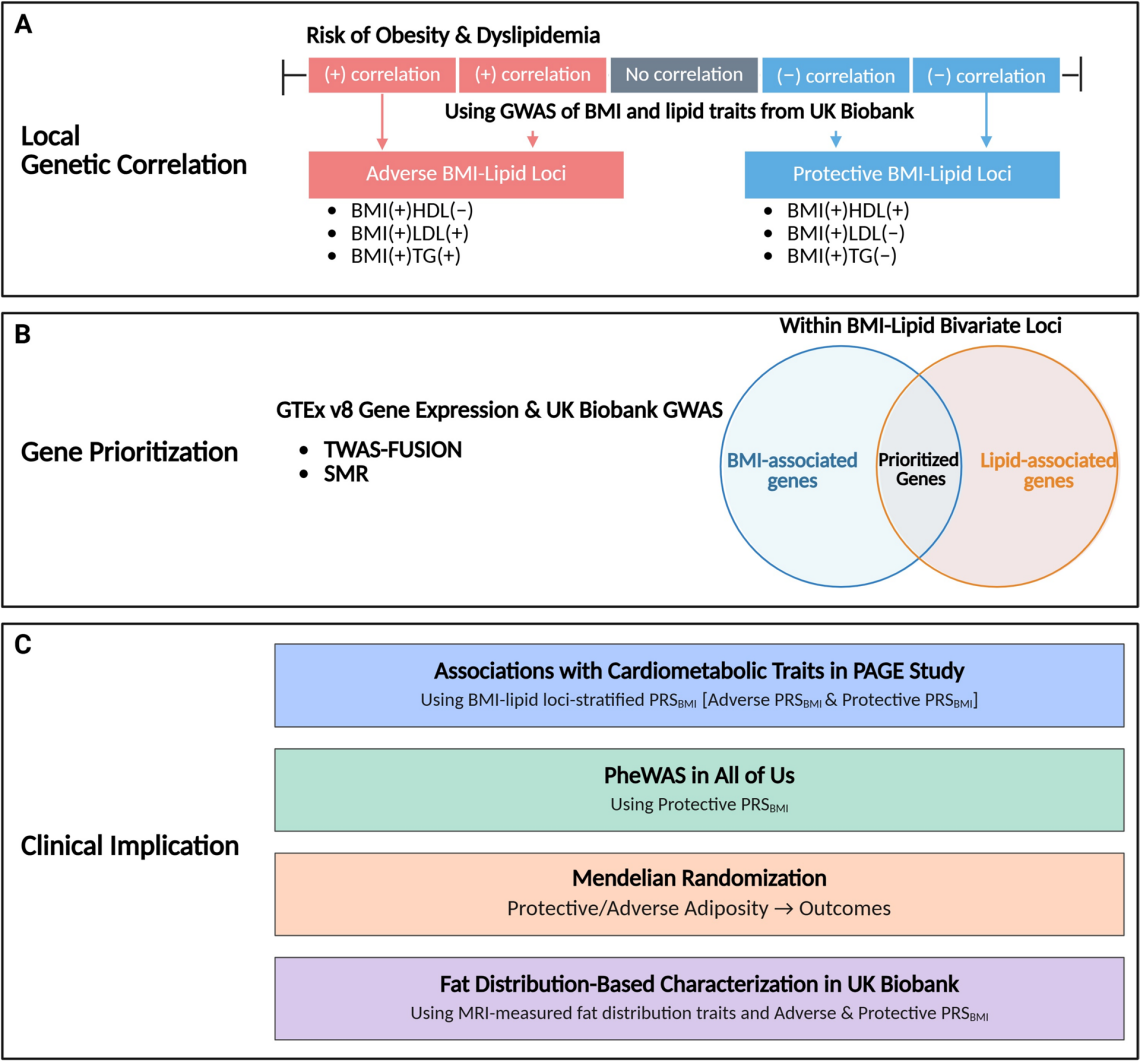
Overview of analytical framework. **A** We first identified pleiotropic genomic loci showing opposite directions of association between BMI and lipid traits using local genetic correlation analyses (implemented through LAVA). **B** To prioritize candidate causal genes within the BMI-lipid bivariate loci, we performed transcriptome-wide association studies (TWAS) using FUSION (TWAS-FUSION) and summary-based Mendelian randomization (SMR). Significant genes were filtered based on consistent associations with both BMI and the corresponding lipid trait in the hypothesized direction. **C** Clinical implications of the protective and adverse BMI-lipid bivariate loci were evaluated through four analyses: (1) association testing of bivariate loci-stratified polygenic risk scores (PRS_BMI_) with cardiometabolic traits in the PAGE study; (2) Phenome-wide association study (PheWAS) in All of Us using PRS_BMI_ based on protective loci only; (3) Mendelian randomization to infer causal relationships between protective or adverse adiposity and health outcomes; and (4) association of bivariate loci-stratified PRS_BMI_ with MRI-measured fat distribution traits in UK Biobank

Based on the UKBB GWAS summary statistics for BMI and lipid traits (HDL, LDL, and TG), we identified BMI-lipid bivariate loci, genomic loci with shared genetic signals between BMI and lipid levels using local genetic correlation analyses (Fig. 1A). In this analysis, a locus is defined as a near LD-independent pre-partitioned genomic region provided by the *LAVA* (Local Analysis of [co]Variant Association) developers [42]. Three pairs of GWAS summary statistics from UKBB (BMI-HDL, BMI-LDL, and BMI-TG) were used as input files, and local Genetic correlation analyses were performed for the 2495 pre-partitioned genomic regions (~ 1.12 Mb per locus on average) using *LAVA (LAVA R package version 0.1.0)* [43]. Detailed descriptions of analyses using *LAVA* are provided in Additional file 1*.*

We classified significant local genetic correlation estimates [*p* < (0.05 / the number of significant univariate loci for both traits)] into two different BMI-lipid bivariate locus groups, adverse or protective, based on their directions of effect with BMI and lipid levels (Table S6). A bivariate locus was considered adverse if it showed a positive local genetic correlation coefficient (r_g_) between BMI and LDL or TG, or a negative local r_g_ between BMI and HDL (BMI( +)LDL( +) locus, BMI( +)TG( +) locus, or BMI( +)HDL(−) locus). Conversely, a bivariate locus was considered protective if it showed a negative local r_g_ between BMI and LDL or TG, or a positive local r_g_ between BMI and HDL (BMI( +)LDL( −) locus, BMI( +)TG( −) locus, or BMI( +)HDL( +) locus). We considered the protective BMI-lipid bivariate loci counterintuitive, as the phenotypic correlations were in opposite directions (i.e., a phenotypic correlation coefficient (r) < 0 for BMI-HDL, r > 0 for BMI-LDL and BMI-TG). Each BMI-lipid pair was tested separately, so there could be overlapping loci for multiple BMI-lipid pairs, even with different directions (e.g., a locus can be adverse for one BMI-lipid pair and protective for another BMI-lipid pair).

#### Gene prioritization for the BMI-lipid bivariate loci

To investigate biological implications and prioritize the genes related to the protective BMI-lipid bivariate loci, we conducted TWAS-FUSION, following the recommended protocol with default settings (http://gusevlab.org/projects/fusion/) [44] and prioritized potential genes whose genetically predicted expression levels were associated with both BMI and a corresponding lipid trait (Fig. 1B). We integrated each GWAS summary result (BMI, HDL, LDL, and TG) with reference gene expression levels and identified genes whose genetically predicted expression levels were significantly associated with BMI or lipid traits (multiple testing corrected by the number of genes tested). Then, we filtered to the protein-coding genes where the whole gene was located within the bivariate loci and identified the overlapping genes from the BMI GWAS-based results and corresponding lipid trait GWAS-based results. We also examined directional consistency by comparing TWAS-FUSION Z scores for BMI and the corresponding lipid trait. For example, we verified if a gene within BMI( +)HDL( +) loci had the same direction of TWAS-FUSION Z-score for both BMI and HDL in the same tissue. Based on the known roles of the overlapping genes (as reported in public databases (e.g., GeneCards [45])), we inferred potential pathways simultaneously influencing BMI and lipid traits.

We additionally implemented the summary-based Mendelian randomization (SMR) method (https://yanglab.westlake.edu.cn/software/smr/) [46], a transcriptome-wide gene-based MR approach that integrates GWAS and eQTL data to prioritize genes whose expression levels may be linked to a trait of interest. The current analysis used eQTL data for More than 15,000 genes from the GTEx database [47]. SMR utilizes the lead cis-eQTL SNP—pre-identified by the developers—for each gene as an instrumental variable (IV) for MR to test for causal effects on the trait of interest. The input GWAS summary statistics were from UKBB European ancestry populations, which were the same as those used in the local genetic correlation analysis. We first identified genes significantly associated with both BMI and the corresponding lipid trait and then selected protein-coding genes located within the protective BMI-lipid loci. Among these, we retained genes whose associations were directionally aligned with our hypothesized effects (i.e., increased BMI and decreased LDL or HDL or increased HDL), consistent with our TWAS-FUSION identification strategy.

Both TWAS-FUSION and SMR analyses were conducted across six relevant tissues from GTEx v8 [48]: five tissues prioritized in a previous study [49]—whole blood (WB), VAT, SAT, liver, and artery—along with skeletal muscle tissue, which was additionally included due to its relevance in body mass and fat metabolism.

#### Associations of BMI-lipid-stratified PRS_BMI_ with cardiometabolic traits in PAGE

We examined the potential influence of the BMI-lipid bivariate loci in ancestrally diverse PAGE participants (Fig. 1C). We hypothesized that the protective and adverse bivariate loci would be involved in distinct biological pathways, linking adiposity with protective and detrimental roles in lipid metabolism, respectively, and that polygenic risk scores for BMI (PRS_BMI_) constructed with variants restricted to the identified bivariate loci would capture the genetic predisposition to distinct subtypes of adiposity. Based on these assumptions, we constructed three protective BMI-lipid bivariate loci-stratified PRS_BMI_—i.e., PRS_BMI(+)HDL(+)_ constructed with the variants within BMI( +)HDL( +) loci, PRS_BMI(+)LDL(−)_ constructed with the variants within BMI( +)LDL( −) loci, and PRS_BMI(+)TG(−)_ constructed with the variants within BMI( +)TG( −) loci—and three adverse BMI-lipid bivariate loci-stratified PRS_BMI_ (PRS_BMI(+)HDL(−)_, PRS_BMI(+)LDL(+)_, and PRS_BMI(+)TG(+)_) by restricting to the variants within corresponding bivariate loci.

We utilized publicly available PRS weights for BMI based on UKBB summary statistics for BMI, estimated using PRS-CS [50] methods with the auto parameter option (validation set is not needed) [51] (PGS Catalog ID: PGS002844). The Pearson correlation between PRS and BMI in the testing sample was 0.321 [51]. The original weights included 1,113,832 SNPs, but for our analysis, we restricted to the variants within the identified BMI-lipid bivariate loci and applied the corresponding weights to our target population, the PAGE study. Variants that were located outside of the bivariate loci were not included in the PRS calculation. The PRS was calculated using the “–score” function in PLINK (version 2.00a3LM) [52, 53]. The number of SNPs included in each BMI-lipid loci-stratified PRS_BMI_ is shown in Table S7.

We then assessed the associations of the bivariate loci-stratified PRS_BMI_ with BMI and obesity status, lipid traits (HDL, LDL, TG, total cholesterol, and dyslipidemia), CVD risk factors (fasting glucose, fasting insulin, homeostatic model assessment for insulin resistance (HOMA-IR), hemoglobin A1c (HbA1c), type 2 diabetes (T2D) status, SBP, DBP, and hypertension), and CVD outcomes (myocardial infarction (MI) and stroke). We hypothesized that higher protective loci-based PRS_BMI_ would be associated with increased BMI or obesity but, counterintuitively, with protective cardiometabolic profiles. Conversely, higher adverse loci-based PRS_BMI_ would be associated with increased BMI and an adverse cardiometabolic profile. We applied linear regression models for continuous outcomes and logistic regression models for binary outcomes. Included covariates were age, sex, ten genetic principal components (PCs) of ancestry, study, genotype panel, and self-reported race/ethnicity as a social construct associated with the social determinants of health, racism, discrimination, and environmental factors [54, 55]. The PRS analyses were conducted in R (version 4.1.0).

#### Phenome-wide association study of BMI-lipid bivariate loci in AoU

We further explored the potential influence of protective loci-based PRS_BMI_ on various groups of disease outcomes in the AoU study (Fig. 1C). We adopted the PheWAS pipeline used in the previous report [56] and hosted on AoU as a demonstration workspace (“Demo—PheWAS Smoking”). For mapping ICD-9 and ICD-10 codes to phecodes, we utilized Phecode map v1.2. In our study, we utilized different covariates compared to Ramirez et al. [56]: age at last code, sex at birth/gender, 10 ancestry PCs, record depth, and visit frequency. Record depth was estimated as the sum of all visits where an observation or condition code appeared in the EHR. Visit frequency was subsequently calculated by dividing the record depth by the length of the EHR. In our primary analysis, we defined a case as having two instances of a phecode and included all ancestry groups. Under this Model, individuals with only one instance of a phecode were excluded as controls. We also excluded phecodes with less than 100 cases. We conducted sensitivity analyses in which disease status was determined by having one or two phecodes. In another sensitivity analysis, we stratified individuals by populations—European (as defined using HGDP/1000G clusters as described above) and African—to mitigate any potential confounding effects of population structure.

#### Causal effects of BMI-lipid bivariate loci on cardiometabolic outcomes

We conducted two-sample Mendelian randomization (MR) analyses to investigate the causal effects of hypothesized protective and adverse adiposity on 17 health outcome traits. Genetic IVs for protective adiposity were defined as independent lead SNPs (LD *r*^2^ < 0.01) associated with BMI at *p* < 5 × 10^−6^ and located within protective BMI-lipid loci (i.e., BMI( +)HDL( +), BMI( +)LDL(–), and BMI( +)TG(–) loci). Similarly, IVs for adverse adiposity were selected from adverse BMI-lipid loci. To reduce weak instrument bias, SNPs with F-statistics < 10 were excluded. The 17 outcome traits were selected based on traits analyzed in the PRS analysis of the PAGE study or closely related phenotypes, supplemented with two additional traits identified through PheWAS, atrial fibrillation, and dementia. These outcomes included the following: obesity (as a confirmatory trait), HDL, LDL, TG, hypercholesterolemia, SBP, DBP, hypertension, fasting glucose, fasting insulin, HbA1c, HOMA-IR, T2D, MI, stroke, atrial fibrillation, and dementia. We utilized publicly available GWAS summary statistics: FinnGen GWAS [57, 58] for atrial fibrillation, dementia, hypercholesterolemia, hypertension, MI, obesity, stroke, and T2D; Global Lipids Genetics Consortium (GLGC) GWAS [59, 60] for HDL, LDL, and TG; Meta-Analyses of Glucose and insulin-related traits Consortium (MAGIC) GWAS [61–64] for fasting glucose, fasting insulin, HOMA-IR, and HbA1c; and Million Veteran Program (MVP) GWAS [65, 66] for DBP and SBP. Detailed data sources for each outcome are provided in Table S8. For the exposure, we used UKBB GWAS summary statistics for BMI, consistent with those used in the local genetic correlation analyses. MR analyses were performed using the *TwoSampleMR* R package (version 0.6.9) [67]. The primary causal estimates were derived using the inverse-variance weighted (IVW) method. Statistical significance is reported using both a Bonferroni-corrected threshold (*p* < 0.05/17) and a nominal threshold (*p* < 0.05). Results meeting either threshold are reported.

To assess robustness and potential violations of MR assumptions, we conducted sensitivity analyses using the weighted median, weighted mode, and MR-Egger methods. The MR-Egger intercept was used to evaluate directional pleiotropy, and MR-PRESSO was applied to detect and correct for outlier variants that could potentially bias the estimates. We further performed multivariable MR (MVMR) analyses to evaluate whether the observed effects of protective or adverse adiposity on outcomes were mediated through corresponding lipid traits. MVMR was implemented using the MVMR R package (version 0.4) [68]. We used the same set of BMI-associated IVs as in the univariable MR analyses and included SNP–lipid trait associations to estimate the direct effect of BMI while accounting for mediation via lipid metabolism. Effect estimates for the lipid traits in the MVMR analysis were obtained from UKBB GWAS summary statistics, which were also used in the local genetic correlation analysis.

#### Implications of BMI-lipid bivariate loci for fat distribution and body composition

We examined the associations between the BMI-lipid loci-stratified PRS and MRI-derived fat distribution traits to gain insight into the regional fat deposition patterns underlying the identified protective and adverse adiposity loci. Specifically, we tested whether PRSs constructed from BMI-lipid bivariate loci, representing genetically inferred protective and adverse adiposity, were associated with fat distribution traits, thereby providing biological context for these loci. We analyzed eight MRI-based fat distribution traits and their corresponding ratio measures, including BMI as a confirmatory outcome. PRSs were constructed following the same approach used in the PAGE study. Briefly, to generate BMI-lipid loci-stratified PRSs, we selected SNPs located within BMI-lipid (i.e., BMI-HDL, BMI-LDL, and BMI-TG loci) from the genome-wide PRS_BMI_ weight List comprised of HapMap phase 3 SNPs. The Maximum number of participants included in the current analysis was 22,988. The number of SNPs included in each PRS is reported in Table S7. We implemented linear regression Models, adjusting for age, sex, ancestry PC 1 to 10, assessment center, and race/ethnicity. All fat-distribution traits were rank-based inverse normal transformed prior to analysis. PRS were standardized to have a mean of 0 and a standard deviation of 1.

## Results

### BMI-lipid bivariate loci identification in UKBB

To identify two distinct types (protective and adverse BMI-lipid bivariate loci) of correlated genomic regions between BMI and three lipid traits (HDL, LDL, and TG), we conducted local genetic correlation analysis for three BMI-lipid pairs (BMI-HDL, BMI-LDL, and BMI-TG) using the UKBB GWAS summary statistics for these traits. Out of 2495 pre-partitioned Genomic regions, 2268 (BMI-HDL), 1018 (BMI-LDL), and 2017 (BMI-TG) loci demonstrated significant local heritability (*p* < 2.00 × 10^−5^) for both BMI and the respective lipid trait and were further tested for the local genetic correlation (Table S9–S10). As such, we identified 789 HDL, 26 LDL, and 494 TG loci with significant local genetic correlation with BMI. The median and inter-quartile range of local r_g_ among the tested regions were − 0.44 (− 0.56, − 0.31) for BMI-HDL, 0.02 (− 0.12, 0.16) for BMI-LDL, and 0.39 (0.25, 0.52) for BMI-TG, which were comparable to those from global genetic correlation estimates (Table S11). Of these, we identified 3 protective BMI-HDL loci (0.4%) [BMI( +)HDL( +) loci; local r_g_ between BMI and HDL > 0], 10 protective BMI-LDL loci (38.4%) [BMI( +)LDL( −) loci; local r_g_ between BMI and LDL < 0], and 8 protective BMI-TG loci (1.6%) [BMI( +)TG( −) loci; local r_g_ between BMI and TG < 0] (Table 1). The remaining BMI-lipid bivariate loci were adverse (786 BMI( +)HDL( −) loci, 16 BMI( +)LDL( +) loci, and 486 BMI( +)TG( +) loci with opposite local r_g_ compared to the protective BMI-lipid bivariate loci), which was more consistent with the observations from the phenotypic correlations. Furthermore, as expected from the high (negative) correlation between HDL and TG, many of the BMI-HDL bivariate loci overlapped with the BMI-HDL and BMI-TG loci—i.e., a total of 400 adverse loci and two protective loci were identified for both BMI-HDL and BMI-TG pairs (Table S10).

**Table 1:** A list of genomic loci with shared genetic signals between BMI and each lipid trait in a protective direction (opposite to the phenotypic correlation)

Locus	Chr	Start position	Stop position	Discovery pair	Prioritized genes^b^	BMI-HDL		BMI-LDL		BMI-TG	
						Local genetic correlation coefficient	*p*-value	Local genetic correlation coefficient	*p*-value	Local genetic correlation coefficient	*p*-value
158	1	205,009,624	205,917,548	BMI-LDL		− 0.57	2.01E-09	−0.57	2.69E-05	0.32	8.46E-04
498	3	87,411,259	88,375,763	BMI-LDL		− 0.61	6.34E-06	−0.67	3.12E-06	0.48	2.72E-03
692^c^	4	102,544,804	104,384,534	BMI-LDL		− 0.75	9.16E-37	−0.47	3.85E-05	0.68	3.08E-12
836	5	73,314,062	74,245,354	BMI-LDL		− 0.52	5.78E-06	−0.56	9.74E-09	0.46	4.58E-04
837	5	74,245,355	75,239,302	BMI-LDL	TWAS: *GCNT4 (*BMI↑ in Artery) SMR: *HMGCR (*BMI↓ in *Skeletal Muscle), ANKDD1B (*BMI↑ in *VAT)*	− 0.72	1.59E-10	−0.69	5.84E-27	0.11	4.26E-01
965^a^	6	32,586,785	32,629,239	BMI-LDL, BMI-TG		− 0.33	8.97E-03	−0.70	5.83E-08	−0.54	1.80E-06
1185	7	98,173,565	99,465,540	BMI-LDL		− 0.46	5.68E-06	−0.38	8.06E-06	0.35	1.62E-03
1246^a^	8	8,064,601	8,589,770	BMI-TG	TWAS: *PRAG1* (BMI↓ in VAT and Artery, BMI↑ in Liver)	− 0.39	7.23E-04	0.15	2.12E-01	−0.48	8.74E-09
1247^a^	8	8,589,771	9,167,795	BMI-TG	TWAS: *ERI1* (BMI↓ in Liver), *CLDN23, MFHAS1* (BMI↑ in Skeletal Muscle), *PPP1R3B* (BMI↑ in Artery) SMR: *PPP1R3B* (BMI↑ in Artery)	0.04	4.60E-01	0.26	1.82E-03	−0.35	2.28E-06
1248^c^	8	9,167,796	9,835,863	BMI-TG		0.19	1.01E-03	0.36	2.45E-06	−0.62	1.95E-11
1249	8	9,835,864	10,478,851	BMI-TG	TWAS: *MSRA* (BMI↓ in Artery)	− 0.02	7.06E-01	0.51	3.99E-05	−0.41	2.33E-08
1251^a^	8	11,466,762	12,296,849	BMI-TG	TWAS: *NEIL2* (BMI↑ in VAT and Artery), *FDFT1* (BMI↑ in VAT, SAT, WB, Artery), *BLK* (BMI↓ in VAT, SAT, WB), *FAM167A* (BMI↑ in SAT, WB)	− 0.10	2.55E-01	N/A	N/A	−0.36	2.27E-06
1351^a,c^	8	125,453,323	126,766,827	BMI-HDL, BMI-LDL, BMI-TG		0.33	1.33E-05	−0.54	1.51E-14	−0.50	7.14E-14
1851^a,c^	12	123,396,635	124,843,768	BMI-HDL, BMI-TG	TWAS: *RILPL2* (BMI↑ in Skeletal Muscle), *DNAH10* (BMI↑ in VAT), *CCDC92* (BMI↑ in SAT, VAT, Liver, WB), *ZNF664* (BMI↓ in WB, BMII↑ in SAT, VAT) SMR: *CCDC92* (BMII↑ in VAT)	0.37	1.59E-08	−0.43	8.63E-05	−0.53	5.92E-12
2135	16	53,393,883	54,866,095	BMI-LDL	TWAS: *FTO* (BMI↑ in Skeletal Muscle)	− 0.56	4.69E-30	−0.73	5.36E-21	0.18	9.98E-03
2351^c^	19	45,040,933	45,893,307	BMI-HDL, BMI-LDL		0.34	9.97E-13	−0.46	1.21E-27	0.21	2.23E-06

*BMI* body mass index, *Chr* chromosome, *HDL* high-density lipoprotein, *LDL* low-density lipoprotein, *SAT* subcutaneous adipose tissue, *SMR* summary-based Mendelian randomization, *TG* triglycerides, *TWAS* transcriptome-wide association study, implemented using the TWAS-FUSION approach, *VAT* visceral adipose tissue, *WB* whole blood

^a^No discordance across different BMI-lipid pairs (i.e., all three BMI-lipid pairs demonstrated protective direction or non-significant correlation)

^b^Genes identified from TWAS-FUSION analysis and SMR

^c^Reported in previous studies

We discovered novel signals for protective BMI-lipid pleiotropy and confirmed previous reports for other protective BMI-lipid pleiotropic signals. We compared the protective bivariate loci results with findings from five previous studies of counterintuitively protective BMI-CVD risk factor pleiotropy [11, 13, 15, 69, 70]. All five studies used variant-based approaches (e.g., multivariate adiposity and cardiovascular traits GWAS). A total of 149 distinct variants located within 104 loci (out of the 2495 genomic regions used for our local genetic correlation analyses) have been reported as obesity variants associated with protective cardiometabolic profiles (Table S12). Although our analyses were locus-based, and it is difficult to directly compare loci and variants, we identified 11 novel protective loci (7 for BMI( +)LDL( −) loci and 5 for BMI( +)TG( −) loci; 1 overlapping locus), which included no previously reported variants. All three BMI( +)HDL( +) loci, three of ten BMI( +)LDL( −) loci, and three of eight BMI( +)TG( −) loci included at least one of the previously identified protective/favorable adiposity variants. Differences observed across studies may be a result of different discovery populations (though some of the prior studies also utilized UKBB) and/or different identification strategies and methods.

There were overlapping protective bivariate loci across multiple BMI-lipid pairs. We identified four protective bivariate loci (Loc1351 in chromosome 8, Loc2351 in chromosome 19, Loc 965 in Chromosome 6, and Loc1851 in chromosome 12) across multiple BMI-lipid pairs. Loc1351 (Chr8:125,453,323–126,766,827) was protective for all three BMI-lipid pairs. Of these four overlapping loci, three included previously reported protective variants, rs2980888 [11] and rs7005992 [13] in Loc1351, rs7133378 [11, 15, 70], rs7973683 [13], and rs863750 [11] in Loc1851, and rs2075650 [11] in Loc2351 (Table 1 and Table S12).

### Gene prioritization by TWAS-FUSION and SMR

To investigate genes involved in the protective BMI-lipid pleiotropy, we performed TWAS-FUSION and SMR, integrating GWAS summary statistics with GTEx v8 gene expression data across six relevant tissues. We prioritized genes whose genetically predicted expression levels were associated with both BMI and lipid traits in directions discordant with phenotypic correlations (Fig. 1B; Table 1; Tables S13–14). TWAS identified genes at several loci, including *GCNT4* (locus 837), *NEIL2, FDFT1, BLK, *and* FAM167A* (locus 1251), *PRAG1* (locus 1246), *ERI1, CLDN23, MFHAS1, *and* PPP1R3B* (locus 1247), and *MSRA* (locus1249), with expression patterns consistent with protective effects. At locus 1851, *RILPL2, DNAH10, CCDC92, *and* ZNF664* were prioritized, consistent with previous studies of [11] protective adiposity. *FTO* at locus 2135 also showed a protective association for BMI-LDL. In parallel, SMR identified 11 genes as potential causal mediators of protective BMI-lipid associations (Table S14). Among these, 6 genes (*GCNT4, MFHAS1, PPP1R3B, NEIL2, FDFT1*, and *CCDC92*) were also detected in TWAS. Four genes, *CCDC92, PPP1R3B, HMGCR*, and *ANKDD1B*, passed the HEIDI test (P_HEIDI_ > 0.05), suggesting the association was not driven by linkage. Notably, *CCDC92*, supported by both TWAS and SMR in our analysis, was previously reported as a protective adiposity gene.

### Associations of BMI-lipid-stratified PRS_BMI_ with cardiometabolic traits in PAGE

**Fig. 2:**
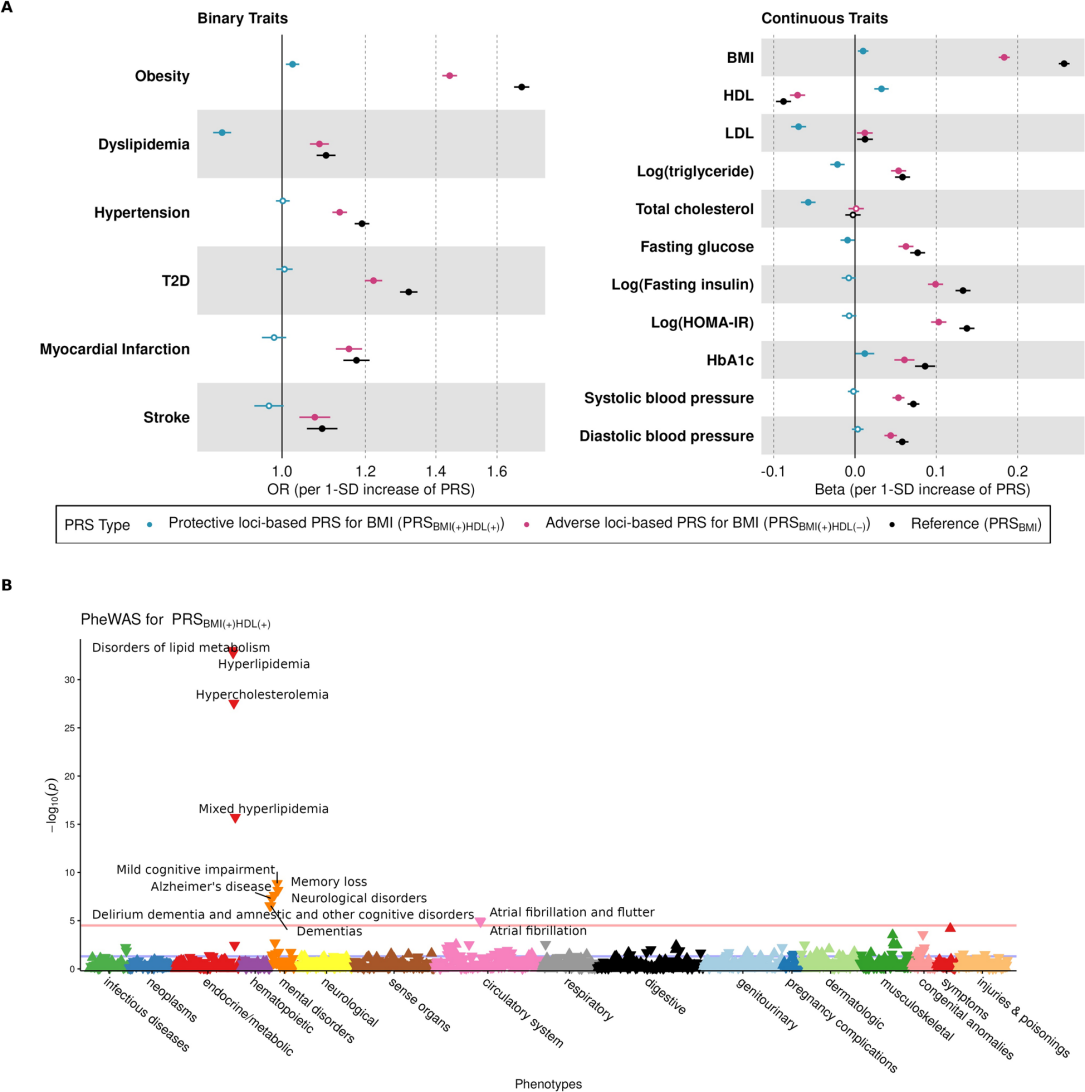
Associations between PRS_BMI(+)HDL(+)_ and cardiometabolic traits in PAGE study. **A** In the PAGE study, PRS_BMI_ constructed using BMI-lipid bivariate loci demonstrated distinct associations with cardiometabolic risk. Specifically, PRS_BMI_ based on protective BMI-HDL loci [PRS_BMI(+)HDL(+)_] was positively associated with BMI and obesity risk but inversely associated with dyslipidemia (higher HDL, lower LDL, TG, and total cholesterol) and fasting glucose. **B** In the All of Us participants, PheWAS analysis of PRS_BMI(+)HDL(+)_ revealed protective associations against lipid-related disorders, atrial fibrillation, and cognitive disorders, including Alzheimer’s disease

To examine the impact of the BMI-lipid bivariate loci in an independent and racially/ethnically diverse population, we tested the associations between PRS_BMI_ stratified by bivariate loci and cardiometabolic outcomes in the PAGE study. Specifically, we examined three protective PRS_BMI_ [PRS_BMI(+)HDL(+)_, PRS_BMI(+)LDL(−)_, and PRS_BMI(+)TG(−)_] and three adverse PRS_BMI_ [PRS_BMI(+)HDL(−)_, PRS_BMI(+)LDL(+)_, and PRS_BMI(+)TG(+)_]. Distinct patterns were observed for BMI-HDL loci: both PRS_BMI(+)HDL(+)_ and PRS_BMI(+)HDL(−)_ were associated with an increased BMI and the risk of obesity, but PRS_BMI(+)HDL(+)_ was linked to a protective CVD risk profile—favorable lipid profiles, lower dyslipidemia prevalence, fasting glucose, and stroke (*p* = 0.08)—whereas PRS_BMI(+)HDL(−)_ showed adverse associations, aligning with the overall association pattern of the reference PRS_BMI_ (Fig. 2A and Table S15–16). For BMI-LDL loci, both PRS_BMI(+)LDL(−)_ and PRS_BMI(+)LDL(+)_ increased BMI and obesity risk, but had mostly similar CVD associations, except PRS_BMI(+)LDL(−)_ was inversely associated with LDL levels (Table S15–S16). Similarly, no clear protective pattern was observed for PRS_BMI(+)TG(−)_. PRS_BMI(+)TG(+)_ demonstrated similar association patterns with the reference PRS_BMI_ (Table S15–S16). The results of the PRS analyses stratified by self-reported race/ethnicity (Table S17–18) and excluding the APOE locus (Table S19–20) are presented in the Additional file 2. These sensitivity analyses were generally consistent with the main findings.

### PheWAS results for the protective BMI-lipid loci-based PRS_BMI_ in AoU

We conducted a PheWAS in the AoU to evaluate the clinical implications of protective PRS_BMI_. Among 1302 distinct disease outcomes tested, 12 diseases from endocrine/metabolic, mental disorders, and circulatory categories were inversely associated with PRS_BMI(+)HDL(+)_ (Fig. 2B and Table S21). These included hyperlipidemia [OR (95% CI) 0.91 (0.89–0.92), *p* = 1.72 × 10^−33^], atrial fibrillation (circulatory) [OR (95% CI) 0.94 (0.91–0.97), *p* = 1.28 × 10^−5^], and cognitive disorders such as Alzheimer’s disease [OR (95% CI) 0.71 (0.63–0.80), *p* = 5.08 × 10^−8^]. We also observed a positive association between the PRS and an adiposity measure, BMI, (*β* (SE) = 0.08 (0.02), *p* = 4.62 × 10^−4^) in a separate regression analysis of non-EHR data. Furthermore, among the other disease outcomes tested in the PAGE study (MI, stroke, T2D, and hypertension), ischemic heart disease (phecode 411) (i.e., MI) [OR (95% CI) 0.97 (0.96–0.99), *p* = 6.70 × 10^−3^] and cerebrovascular disease (phecode 433) (i.e., stroke) [OR (95% CI) 0.97 (0.95–0.99), *p* = 1.58 × 10^−2^] were nominally associated with the PRS in a protective direction (Table S21). In the sensitivity analysis using a single phecode definition, no meaningful differences in associations were observed (Table S22). Since the protective BMI-HDL loci included the *APOE* locus known for strong pleiotropy, in another sensitivity analysis, we excluded the variants within the *APOE* locus from the PRS calculation. As a result, protective associations with metabolic and circulatory diseases persisted, but the association with dementia disappeared (Table S23). This suggests that the apparent protective effect on dementia is driven by the *APOE* locus, while the cardiometabolic effects reflect broader polygenic architecture.

For the other two protective PRS_BMI_ [PRS_BMI(+)LDL(−)_ and PRS_BMI(+)TG(−)_], we did not observe the hypothesized protective associations in AoU (Tables S24–26, S27–28), which is consistent with the results in PAGE. PRS_BMI(+)LDL(−)_ was associated with diseases from multiple categories, including endocrine/metabolic, circulatory, and musculoskeletal; however, the direction of effect for each was non-protective, as opposed to our hypothesis, and more aligned with overall PRS_BMI_ reported from the previous PheWAS studies [71, 72].

### Putative causal effects of BMI-lipid bivariate loci on cardiometabolic outcomes (MR results)

**Fig. 3:**
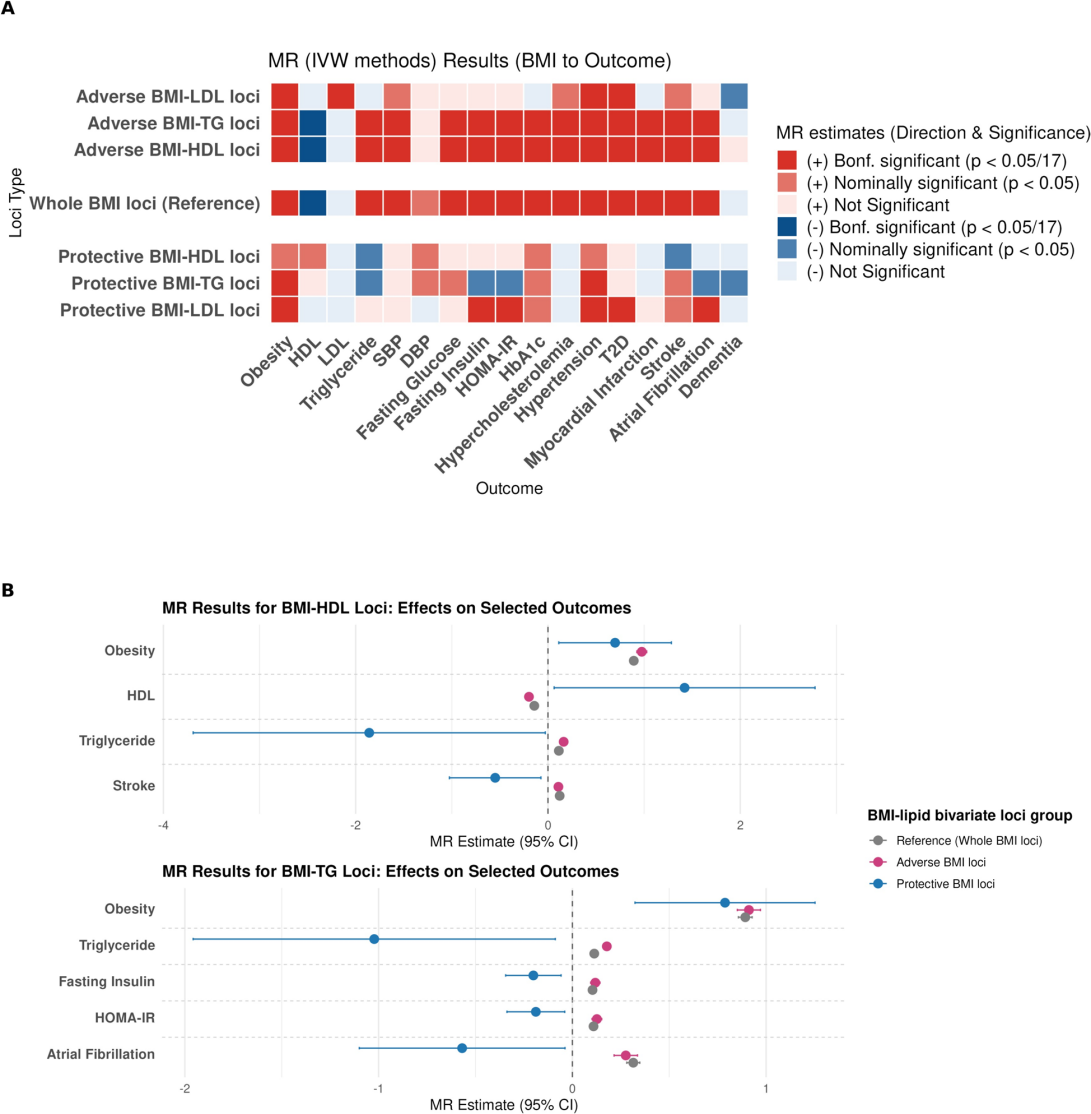
Mendelian randomization (MR) results for BMI–lipid bivariate loci and cardiometabolic traits. **A** MR was conducted using BMI-associated SNPs from protective and adverse loci separately for 17 cardiometabolic traits. **B** Protective BMI–HDL loci showed nominal associations with higher HDL, lower TG, and reduced risk of stroke. Protective BMI–TG loci were nominally associated with lower TG, fasting insulin, HOMA-IR, and reduced risk of atrial fibrillation. Adverse loci showed directionally opposite associations, supporting etiologic heterogeneity

To further assess the impact of BMI-lipid bivariate loci, we conducted MR analyses using BMI-associated SNPs from protective and adverse loci separately. The MR results showed a clear contrast between protective and adverse BMI-lipid loci, particularly in BMI-HDL and BMI-TG loci, in terms of their causal associations with cardiometabolic traits. For BMI-HDL loci, IV SNPs for BMI from the protective and adverse loci demonstrated opposite directional effects, with nominally significant associations observed for HDL, TG, and stroke, each consistent with the hypothesized protective or adverse effects (Fig. 3A and B). Similarly, for BMI-TG loci, protective and adverse loci showed significantly opposing effects on TG, fasting insulin, HOMA-IR, and atrial fibrillation, again supporting our hypothesis (Fig. 3A and B). In addition, while IV SNPs for BMI within the protective BMI-TG loci were nominally protective against dementia, those within the adverse BMI-TG loci, like the full set of BMI loci (reference), showed null associations with dementia. Notably, the protective BMI-TG did not include the *APOE* locus, suggesting this dementia-related association is independent of *APOE*. Adverse BMI-HDL and BMI-TG loci showed causal association patterns with cardiometabolic traits that were highly similar in both direction and significance to the reference BMI loci. In contrast, neither the protective nor the adverse BMI-LDL loci displayed the hypothesized contrast, echoing the lack of consistent patterns observed in the PRS and PheWAS results (Fig. 3A and B, Table S29).

To investigate whether lipid traits mediated the effects of protective or adverse BMI loci on cardiometabolic outcomes, we conducted MVMR analyses by including the corresponding lipid trait (HDL or TG) as an additional explanatory variable for outcomes that showed opposite and significant associations (*p* < 0.05) in univariate MR (Table S30). After adjusting for the corresponding lipid, none of the protective BMI loci remained significant. This suggests that the protective associations observed in univariate MR may be mediated through HDL or TG biology, rather than reflecting lipid-independent effects of adiposity on these outcomes.

### Implications of BMI-lipid bivariate loci for fat distribution and body composition

**Fig. 4:**
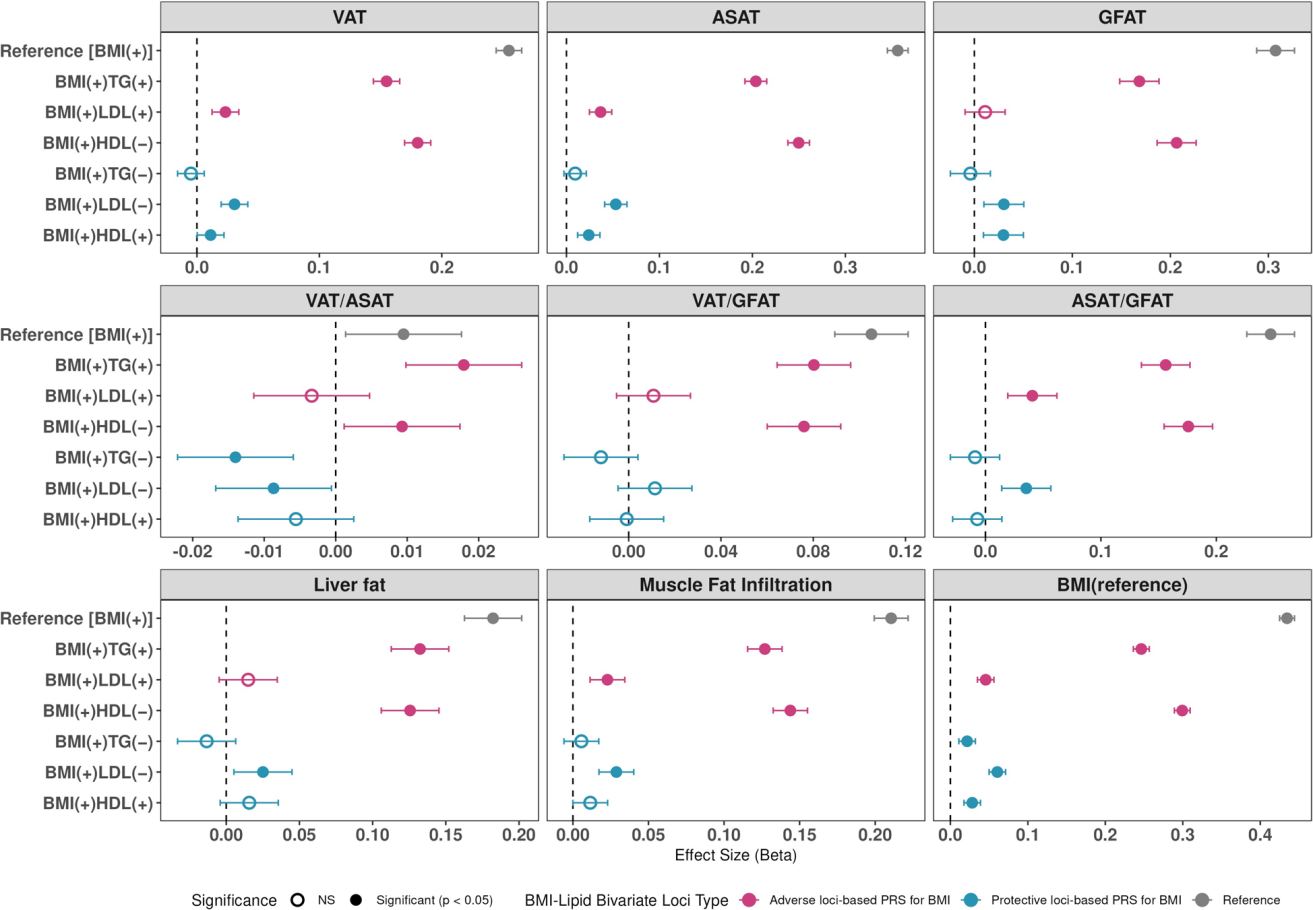
Characterization of BMI-lipid loci using MRI-derived regional fat measures. Associations between bivariate loci-stratified PRS_BMI_ and MRI-based fat distribution traits in the UKBB revealed that the PRS_BMI_ based on protective BMI-TG loci was uniquely and inversely associated with the VAT/ASAT, suggesting reduced visceral fat relative to abdominal subcutaneous fat. No such pattern was observed for other PRS_BMI_

PRSs effect estimates for absolute fat volumes (VAT, ASAT, and GFAT) largely mirrored those of PRS_BMI_, consistent with their strong correlation with overall adiposity as captured by BMI (Fig. 4 and Table S31). In contrast, PRSs effects on ratio measures revealed distinct patterns. In particular, both protective PRS_BMI(+)TG(−)_ and PRS_BMI(+)LDL(−)_ were inversely associated with VAT/ASAT. However, among the corresponding adverse PRSs, only PRS_BMI(+)TG(+)_ showed a positive association with VAT/ASAT, whereas PRS_BMI(+)LDL(+)_ was not significantly associated. Taken together, these findings suggest that BMI-TG loci-stratified PRS may influence the relative distribution between VAT and ASAT.

## Discussion

In this study, using large-scale GWAS summary statistics derived from the UKBB, we identified 16 genomic regions with shared genetic underpinnings between BMI and Lipid levels, which were associated with increased obesity risk but decreased risk for dyslipidemia. We further explored the potential causal Genes underlying the protective BMI-lipid bivariate loci using Gene-based TWAS-FUSION and SMR results and prioritized 18 compelling candidate genes for further consideration. Using bivariate loci-stratified PRS_BMI_, specifically PRS_BMI(+)HDL(+)_, we observed protective associations with lipid-related traits, CVD, and cognitive disorders in independent populations. MR further supported a distinct causal link between protective adiposity (measured through BMI-associated SNPs within the protective BMI-lipid loci) and decreased risk of cardiometabolic outcomes such as atrial fibrillation and stroke. Finally, associations with MRI-based fat distribution measures suggested that these protective adiposity loci may reflect a phenotype characterized by reduced visceral to abdominal subcutaneous fat ratio.

In the loci identification stage using UKBB GWAS summary statistics, the smaller global genetic correlations between BMI and LDL in comparison to BMI-HDL and BMI-TG have been consistently reported in the literature [73, 74]. By performing local-level genetic correlation analysis for BMI and LDL, we investigated whether there is a true lack of genetic correlation (both locally and globally) between BMI and LDL or whether the lack of global genetic correlation is due to the presence of local-level correlations in opposite directions that globally nullify the effects of the other regions. The current study supported both possibilities—(i) a much smaller number of BMI-LDL correlated loci was identified, implying a lack of genetic correlation as compared to the BMI-HDL or BMI-TG pairs, and (ii) the numbers of protective [BMI( +)LDL( −)] and adverse [BMI( +)LDL( +)] loci are comparable. Indeed, many more adverse bivariate loci were discovered compared to protective loci for BMI-HDL and BMI-TG, as expected from the high phenotypic positive correlation between obesity and dyslipidemia [75]. It is also true that, unlike BMI-HDL or BMI-TG results, a similar number of adverse loci and protective loci were identified among BMI-LDL, and they might have nullified each other’s effects, resulting in a small magnitude of global genetic correlation between BMI and LDL. These differences in BMI-lipid pairs (BMI-TG, BMI-HDL vs. BMI-LDL) may suggest the presence of distinct obesity-lipid inter-relationships for HDL and TG versus LDL [76, 77].

By integrating TWAS-FUSION and SMR results with the current local genetic correlation analysis, we prioritized potential causal genes, both novel and known genes, underlying the counterintuitively protective genetic correlations between BMI and lipid traits. As an example of the plausible novel genes, we prioritized the *NEIL2* gene within the BMI( +)TG( −) locus (Loc1251). *NEIL2*, Nei-like DNA Glycosylase 2, is involved in Autosomal Dominant Adult-Onset Proximal Spinal Muscular Atrophy [78] which is relevant for both reduced body weight and an adverse CVD risk profile. According to the GWAS catalog, variants in/near the *NEIL2* gene have been associated with TG levels and waist-to-hip ratio adjusted for BMI and other CVD traits, further supporting the *NEIL2* gene as a potential causal gene influencing adiposity and CVD. The *FDFT1* gene in the same locus was also a plausible candidate. As *FDFT1* encodes squalene synthase, it is closely related to cholesterol synthesis, and high serum squalene levels have been associated with abdominal obesity [79]. Furthermore, another gene identified in the locus, *BLK*, is associated with maturity-onset diabetes of the young (MODY), a subtype of diabetes. In Loc1247, the *PPP1R3B* gene was prioritized by both TWAS-FUSION and SMR. It is known to play a crucial role in liver glycogen metabolism [80] and may influence lipid metabolism through this pathway. As consecutive loci from Loc1246 to Loc1251, except for Loc1250, were identified as protective BMI-TG bivariate loci, the genomic loci spanning Loc1246 to Loc1251 may harbor protective adiposity loci. The fact that Loc1248 included known protective adiposity variants further supported the current finding. In addition, *FTO*, a novel potential causal gene within the BMI( +)LDL( −) locus (Loc2135), is a well-established obesity-associated gene, and BMI-increasing risk alleles of the SNPs in *FTO* have been associated with an adverse cardiometabolic profile [81–83]; however, some previous studies reported paradoxically favorable influences of *FTO* on cardiometabolic risk profiles [84, 85]. We also identified *HMGCR* as one potential causal gene for protective BMI-LDL loci. *HMGCR* is a pharmaceutical target of statins—i.e., inhibition of HMGCR reduces cholesterol synthesis, thereby lowering blood cholesterol levels [86]. Although its role in body weight or adiposity is not fully understood, a previous study reported an association between a SNP in *HMGCR* and LDL and body weight in opposite directions [87]. As described above, the genes prioritized in this study are each directly or indirectly related to adiposity and lipid metabolism, supporting their potential roles as causal genes. However, further studies are needed to complete functional follow-up and elucidate the mechanisms by which these genes exert their protective influence on lipid and cardiometabolic traits.

In addition to novel genes, several of our findings strengthen the findings from previous studies. For example, in Loc1851, we identified three genes (*ZNF664, DNAH10*, and *CCDC92*) associated with BMI, TG, and HDL. Notably, *CCDC92* was prioritized by both TWAS-FUSION and SMR analyses. *ZNF664, DNAH10*, and *CCDC92* have previously been prioritized as candidate causal genes for the adiposity variants associated with a protective cardiometabolic profile [11]. Indeed, *DNAH10* and *CCDC92* have been identified as insulin resistance-related genes, and gene knockdown experiments have supported their associations with the decreased peripheral adipose deposition capacity [13].

Our findings of heterogeneous BMI-lipid bivariate loci support a potential clinical utility of the stratified PRS_BMI_. From the BMI-HDL bivariate loci, we provide evidence of a generalizable heterogeneous genomic relationship between obesity and dyslipidemia. Notably, in the PAGE study, the increase in PRS_BMI(+)HDL(+)_ was associated with protective lipid values (lower risk of dyslipidemia), while it was associated with increased BMI and increased risk of obesity. These results suggest that shared genetic underpinnings between obesity traits and lipid traits may partly explain the heterogeneous impact of BMI on CVD risk. Of note, despite the small number of variants included in the PRS_BMI(+)HDL(+)_—i.e., ~ 1500 variants (from three loci) in PRS_BMI(+)HDL(+)_ vs. ~ 1.11 M variants in overall PRS_BMI_ and ~ 373 K variants in PRS_BMI(+)HDL(−)_, its effect size on dyslipidemia was greater than that of from overall PRS_BMI_ and PRS_BMI(+)HDL(−)_ in opposing directions.

PheWAS findings provide an exhaustive list of disease outcomes associated with the protective PRS_BMI_, which is distinct from the associations with overall PRS_BMI_ or phenotypic association with BMI. First, the PheWAS results confirmed the protective associations of PRS_BMI(+)HDL(+)_ with lipid metabolism and CVDs (lower risk for atrial fibrillation with Bonferroni-corrected significance and ischemic heart disease and cerebrovascular disease with nominal significance) as observed in the PAGE study. This is in contrast to a previous phenome-wide Mendelian randomization (MR) study [71] where overall PRS_BMI_ was adversely associated with lipid-related disorders—e.g., hyperlipidemia [OR (95% CI) 1.47 (1.41–1.54)], atrial fibrillation [OR (95% CI) 1.61 (1.52–1.71)], and ischemic heart disease [OR(95% CI) 1.67 (1.60–1.74)]. The elevated risk of the abovementioned CVDs with the increase in PRS_BMI_ has also been reported in eMERGE [72]. Thus, our results support the presence of distinct adiposity-associated genetic loci heterogeneously influencing CVD risk.

In addition, we observed protective associations of PRS_BMI(+)HDL(+)_ with several cognitive disorders, such as mild cognitive disorders, memory loss, Alzheimer’s disease, and dementia. The phenotypic association between BMI and cognitive disorders has been inconclusive; some studies reported that lower BMI was associated with an increased risk of Alzheimer’s disease [88–90], whereas other study reported obesity and overweight as a risk factor for dementia [91]. To our knowledge, most MR studies did not find evidence of any association between genetically influenced BMI and dementia [71, 92, 93] or reported a positive association between genetically predicted BMI and Alzheimer’s disease [94]. Unlike previous studies based on overall BMI genetics, the current findings suggest that specific local-level BMI genetics may play a protective role against cognitive disorders, potentially mediated through HDL levels, contrasting with global-level BMI genetics. The current results may suggest potential roles of favorable adiposity in protecting against cognitive disorders, which may be reflected in the often-reported inverse phenotypic association between BMI and cognitive disorders. We also note that our PRS_BMI(+)HDL(+)_ includes the well-known lipid- and dementia-associated *APOE* locus. In a sensitivity analysis excluding the APOE locus from the PRS, the association with dementia is no longer observed. Thus, the observed protective effects of the PRS_BMI(+)HDL(+)_ on dementia risk are likely driven by *APOE* variants. Further studies are warranted to replicate the findings and elucidate the underlying biological mechanisms.

MR analyses highlight a distinct causal relationship between BMI-associated SNPs within the protective BMI-HDL and BMI-TG loci and favorable cardiometabolic outcomes, including increased HDL, decreased TG, fasting insulin, and HOMA-IR, and reduced risk of stroke and atrial fibrillation. In contrast, BMI-associated SNPs within the adverse BMI-HDL and BMI-TG loci showed significant associations with these conditions in the opposite direction. These findings also contrast with traditional MR results using genome-wide BMI-associated SNPs, as well as our reference analysis, in which BMI SNPs were generally associated with adverse cardiometabolic profiles [95–97]. Further MVMR analyses suggested that the protective effects on these cardiometabolic conditions appear to be mediated through HDL or TG. Together with PRS analyses and PheWAS findings, our MR results support the existence of heterogeneous forms of adiposity, some with protective and others with adverse downstream cardiometabolic effects. Our findings suggest that the identified loci may contribute to the genetic architecture underlying protective adiposity.

Using MRI-based fat distribution measures, we observed a distinct inverse association between protective PRS_BMI(+)TG(−)_ and VAT/ASAT, compared to both the reference PRS_BMI_ and adverse PRS_BMI(+)TG(+)_ groups. This unique association suggests that the protective adiposity captured by BMI-TG loci may reflect a lower proportion of VAT, which is considered metabolically detrimental [98], relative to ASAT. These findings suggest that the identified protective adiposity loci may contribute to heterogeneous fat distribution patterns, consistent with prior evidence indicating that the cardiometabolic consequences of adiposity depend on the location of fat depots [99].

The present study has some limitations. First, the genomic partitioning was based on a European-ancestry LD structure (1000 Genomes European population [25]); thus, the partitioned genomic regions may not apply well to non-European ancestry populations. We may, therefore, have missed ancestry-differentiated genetic correlations. In addition, since BMI is a crude proxy for adiposity, our approach may have missed important pleiotropic loci that link obesity to lipid traits. However, BMI is widely used and readily measured in large-scale cohorts, allowing for substantially larger sample sizes. These larger sample sizes, in turn, substantially increase statistical power to detect genetic associations, particularly for variants with modest effect sizes. This enhanced power helps to identify robust signals that may be relevant to more refined obesity-related traits. Thus, the gains in power afforded by large sample sizes may help to offset the limitations related to its lack of specificity in capturing diverse aspects of adiposity. Nonetheless, we acknowledge that alternative anthropometric measures such as waist-to-hip ratio or waist circumference, as well as imaging-based adiposity measures, may better capture fat distribution and its metabolic consequences. Future studies incorporating these more specific indices will be important to validate and extend our current findings.

The current study has notable strengths as well. First, the total sample size of the PAGE study was large, and we were able to evaluate the relationships between BMI-lipid bivariate loci and various CVD profiles with individual-level phenotype data. In addition, the distribution of self-identified race/ethnicity in the PAGE study, especially across White, Black, and Hispanic/Latino self-identified populations, was well-balanced, thus equally contributing to the population-pooled results. Furthermore, this study implemented a novel locus-based approach to identify BMI-lipid bivariate loci and proposed a novel application of locus-restricted PRS to evaluate the influence of certain genomic loci on phenotypes. We were also able to leverage an independent, large, and diverse US population (All of Us) for PheWAS analyses.

## Conclusions

We identified two distinct types of BMI-lipid bivariate loci in opposing directions (protective versus adverse) and suggested potential causal genes (e.g., *NEIL2, FDFT1, or BLK)* underlying protective BMI-lipid loci. Notably, the bivariate loci-stratified PRS_BMI_, specifically PRS_BMI(+)HDL(+)_, provided evidence of protective influences on lipid-related traits, CVD, and cognitive disorders in independent populations. MR analyses further supported causal relationships between protective BMI loci and multiple cardiometabolic outcomes. The current findings suggest the presence of heterogeneous obesity-related genetics at the local level, which cannot be observed at global-level obesity genetics. With much larger sample sizes, disease-risk stratification by integrating both protective PRS_BMI_ and adverse PRS_BMI_ could be clinically meaningful. Specifically, individuals with comparable genetic risk for overall adiposity can be further stratified based on their genetic predisposition to metabolically favorable and unfavorable adiposity. This stratification may enable more tailored clinical monitoring or preventive interventions, for example, prioritizing intensive lipid management or early lifestyle interventions in individuals with a high genetic predisposition to adverse adiposity or a low genetic predisposition to protective adiposity.

## Supplementary Information

Additional file 1: Supplementary methods. Detailed description of the methods used in this study. Includes: (1) overview of PAGE-participating cohort studies, (2) phenotype definitions and measurement procedures, (3) cardiovascular disease ascertainment protocols used across PAGE studies, (4) GWAS of BMI and lipid traits in the UKBB, and (5) analytical framework for local genetic correlation analysis.

Additional file 2: Supplementary Tables.

## Data Availability

The UKBB GWAS summary statistics for individuals of European ancestry used in the current study for the local genetic correlation analysis were publicly available at https://pan.ukbb.broadinstitute.org/downloads [19, 20] .Specifically, phenocode 21001 for BMI, phenocode 30760 for HDL, phenocode 30780 for LDL, and phenocode 30870 for TG were utilized. The UKBB also provides open access to researchers, who can request access to the dataset by registering and submitting an application at http://ukbiobank.ac.uk/register-apply/ [27]. The PAGE datasets are available through dbGaP [https://www.ncbi.nlm.nih.gov/gap; study accession phs000356] [22, 100]. The All of Us data used in the current study is available to registered researchers of the All of Us Researcher Workbench (for more information: https://www.researchallofus.org/) [101]. GWAS summary statistics used in the MR analyses are publicly available at https://www.finngen.fi/en/access_results for FinnGen [57, 58], https://csg.sph.umich.edu/willer/public/glgc-lipids2021/results/trans_ancestry/ for GLGC [59, 60], http://magicinvestigators.org/downloads/ for MAGIC [61–64], and https://www.ebi.ac.uk/gwas/publications/39024449 for MVP [65, 66]. All results from this study are presented within the main article and its supplementary materials.

## Abbreviations

AoU: All of Us
ARIC: Atherosclerosis Risk in Communities
ASAT: Abdominal subcutaneous adipose tissue volume
BMI: Body mass index
CARDIA: Coronary Artery Risk Development in Young Adults Study
CVD: Cardiovascular disease
DBP: Diastolic blood pressure
EHR: Electronic health records
GLGC: Global Lipids Genetics Consortium
GWAS: Genome-wide association study
HbA1c: Hemoglobin A1c
HCHS/SOL: Hispanic Community Health Study / Study of Latinos
HDL: High-density lipoprotein cholesterol
HGDP: Human Genome Diversity Project
HOMA-IR: Homeostatic Model Assessment for Insulin Resistance
LAVA: Local Analysis of [co]Variant Association
LD: Linkage disequilibrium
LDL: Low-density lipoprotein cholesterol
MAGIC: The Meta-Analyses of Glucose and Insulin-related traits Consortium
MEC: Multiethnic Cohort Study
MEGA: Multi-Ethnic Genotyping Array
MI: Myocardial infarction
MR: Mendelian Randomization
MVP: Million Veteran Program
PAGE: Population Architecture using Genomics and Epidemiology
PC: Principal component
PheWAS: Phenome-Wide Association Study
PRS: Polygenic risk score
r_g_: Genetic correlation coefficient
SAT: Subcutaneous adipose tissue
SBP: Systolic blood pressure
SNP: Single nucleotide polymorphism
T2D: Type 2 diabetes
TG: Triglycerides
UKBB: UK Biobank
VAT: Visceral adipose tissue
WB: Whole blood
WGS: Whole genome sequencing
WHI: Women’s Health Initiative
