# Supplementary material for: Genetic underpinnings of the heterogeneous impact of obesity on lipid levels and cardiovascular disease
Source: Genome Med. 2025 Oct 6;17:113. doi: 10.1186/s13073-025-01522-9 (PMC12502452; doi:10.1186/s13073-025-01522-9)
Supplement: Supplementary file 1 — Additional file 1: Supplementary methods. Detailed description of the methods used in this study. Includes: (1) overview of PAGE-participating cohort studies, (2) phenotype definitions and measurement procedures, (3) cardiovascular disease ascertainment protocols used across PAGE studies, (4) GWAS of BMI and lipid traits in the UKBB, and (5) analytical framework for local genetic correlation analysis. [file 13073_2025_1522_MOESM1_ESM.docx]

**SUPPLEMENTARY INFORMATION**

**PAGE-participating cohort studies**

**ARIC**, funded by the National Heart, Lung, and Blood Institute (NHLBI), is an ongoing community-based prospective cohort study primarily aiming to investigate the etiology of atherosclerosis and its clinical outcomes.^1^ A random sample of 15,792 adults aged 45 – 64 years at baseline was initially recruited between 1987 and 1989 (approximately 4,000 participants for each of four communities in the U.S. – Forsyth County, NC; Jackson, MS; Washington County, MD; Minneapolis, MN).^1^ Participants have received standardized examinations on their demographic, social, and health status approximately every five years.

**BioMe**, funded by The Charles Bronfman Institute for Personalized Medicine, is an electronic medical record-linked biobank whose participants were based on consented and volunteered patients in the Mount Sinai Medical Center (MSMC) (among over 70,000 inpatients and 800,000 outpatients annually).^2^ The MSMC serves racially/ethnically diverse communities of upper Manhattan area, which includes Central Harlem (predominantly African American), East Harlem (predominantly Hispanic/Latino), and Upper East Side (predominantly European American). There have been more than 57,843 participants (21% African American, 34% Hispanic/Latino, 31% European American, and 14% of other ancestry groups) enrolled in BioMe since 2007 (as of Feb 2021). Among them, a total of 32,344 participants have been genotyped (as of Feb 2021), so that they can be investigated in genomic studies (<https://icahn.mssm.edu/research/ipm/programs/biome-biobank/facts>).

**CARDIA**, funded by NHLBI, is a community-based prospective cohort study aiming to investigate the influencing factors for the development of coronary heart disease and its risk factors during young adulthood.^3^ Initial recruitment was done in 1985 – 1986, and a total of 5,116 African American (52%) and European American (48%), aged 18 – 30 years, participated from four urban communities – 1,179 from Birmingham, AL; 1,109 from Chicago, IL; 1,402 from Minneapolis, MN; and 1,426 from Oakland, CA.^3^ In the recruiting step, participants were selected for the cohort to be balanced in its age (> or ≤ 24 years), educational level (> or ≤ 12 years), sex, and race/ethnicity.^3^ After the initial examination, participants were asked to respond to the follow-up examinations during 1987 – 1988 (Year 2), 1990 – 1991 (Year 5), 1992 – 1993 (Year 7), 1995 – 1996 (Year 10), 2000 – 2001 (Year 15), 2005 – 2006 (Year 20), 2010 – 2011 (Year 25), and 2015 – 2016 (Year 35) (and currently Year 40 exam is ongoing as of Dec 2022). Data collection included the potential influencing factors for coronary heart disease – e.g., blood pressure, glucose levels, blood cholesterol levels, anthropometric traits, lifestyle factors, and family history.

**HCHS/SOL**, funded by NHLBI and other institutes, is a community-based prospective cohort study of Hispanic/Latino populations in the U.S. aiming to determine the role of acculturation in the prevalence and incidence of diseases and to identify influencing factors for the health of Hispanic/Latino populations. A total of more than 16,000 participants who were self-identified as Hispanic/Latinos and aged 18 – 74 years were recruited between 2008 and 2011 from four study sites – Bronx, NY; Chicago, IL; Miami, FL; and San Diego, CA. The study was designed to enroll 4,000 participants (2,500 aged 45 – 74 years and 1,500 aged 18 – 44 years) in each study site and to have at least 2,000 participants in each of the four groups of origin – Cuban, Puerto Rican, Mexican, or Central/South American.^4^ The participants received extensive baseline examinations on psych-social and clinical factors during 2008 – 2011. A follow-up assessment for the cohort was done during 2015 – 2017, the third exam is in progress now and annual follow-up interviews via phone calls are ongoing.

**MEC**, funded by the National Cancer Institute, is a prospective cohort study to investigate lifestyle and genetic risk factors for cancer in the U.S. ^5^ A total of 215,251 adults aged 45 – 75 years at baseline were recruited between 1993 and 1996 from Hawaii and L.A. County, CA.^5^ Ethnic distributions of the participants were 16.3% of African American, 22.0% of Hispanic/Latino, 26.4% of Japanese American, 6.5% of Native Hawaiian, 22.9% of European American, and 5.8% of other ethnic groups.^5^ During 2001 – 2006, a prospective biospecimen collection (i.e., biospecimen collected before the onset of disease; blood, urine, mouthwash, saliva, or viable lymphocytes) was done for a subset of participants (75,928 as of April 2019) (https://www.uhcancercenter.org/for-researchers/mec-cohort-composition). In this proposal, eight ancillary studies will be included – the Slim Initiative in Genomic Medicine for the Americas (MEC-Sigma) (a type 2 diabetes study in Hispanic/Latino adults); MEC-AAPC, MEC-JAPC, and MEC-LAPC (studies of prostate cancer in African American, Japanese American, and Hispanic/Latino men, respectively); MEC-AABC, MEC-JABC, MEC-LABC, and MEC-HIBC (studies of breast cancer in African American, Japanese American, Hispanic/Latino women, and Native Hawaiian women, respectively).

**WHI**, funded by NHLBI, is a prospective cohort study to investigate the health of postmenopausal women in the U.S., especially for preventing CVD, breast cancer, colon cancer, and osteoporotic fractures in women aged 50 – 79 years.^6^ A total of 161,808 participants were recruited between 1993 and 1998 at 40 clinical centers across the U.S. There are two different parts in WHI – one is the WHI Clinical Trial (~64,500), a randomized clinical trial of hormone therapy, dietary intervention, and calcium/vitamin D supplements, and the other is WHI Observational Study (~100,000), investigating incidence, risk factors, and potential interventions for CVD, cancer, and osteoporotic fractures.^6^ Followings are ancillary studies that will be included in our analyses – the Genetics and Epidemiology of Colorectal Cancer Consortium (GECCO); the Modification of PM-Mediate Arrhythmogenesis in Population study (MOPMAP); the Genomics and Randomized Trials Networks (GARNET); the Hip Fracture GWAS (HIPFX); the Long Life Study (LLS); the Women’s Health Initiative Memory Study (WHIMS); and the Women’s Health Initiative-SNP Health Association Resource (WHI-SHARe).

**Phenotype measurement**

**BMI.** We used BMI as a continuous proxy measure of obesity risk. BMI was derived from weight and height measured at the baseline visit (at the time of enrollment) for ARIC, BioMe Biobank, CARDIA, HCHS/SOL, and WHI. For 140 WHI participants who were missing in height or weight at baseline, height and/or weight measures at 1-year or 3-year follow-up substituted the missing baseline measures.^7^ In MEC, height and weight measures were self-reported, and these self-reported baseline height and weight measures were used to generate BMI at baseline.

**Lipid traits.** We used three lipid measures as continuous proxies of dyslipidemia risk**.** HDL-C and TG levels were measured from fasting blood, and the Friedewald Equation was used to calculate LDL-C levels from other lipid measures. If measured TG levels were greater than 400mg/dL, LDL-C levels were not calculated. In addition, following previous studies, medication status was handled by adding a constant to each lipid level value according to medication type (**Table below**).^8,9^ The largest constant was applied if more than one medication was reported. Those who had not fasted for 8 hours or were pregnant at measure were excluded from the harmonized phenotype database. Natural-log transformation was applied to TG levels after adjusting for medication. We defined participants as having dyslipidemia if at least one of the three lipid values (total cholesterol, LDL, or triglycerides) was classified as ‘high’ or if the HDL value was classified as ‘low’. Cut-off values for each lipid trait were based on clinical guidelines.^10^

**Constants used for medication adjustment of lipid levels in the PAGE study.**

| Medication | Constants (mg/dL) | | |
| --- | --- | --- | --- |
|  | HDL | LDL | TG |
| Statins | -2.3 | 49.9 | 18.4 |
| Fibrates | -5.9 | 40.1 | 57.1 |
| Bile acid sequestrants | -1.9 | 40.5 | 0 |
| Niacin | -9.9 | 24.7 | 89.4 |
| Cholesterol absorption inhibitors | 0 | 40.5 | 0 |

Source: ^9^

**Glycemic traits**. Fasting blood glucose levels and insulin levels were measured at baseline visits using standard assays after 8 hours of fasting. HbA1c levels were measured during follow-up visits for all cohort studies except for HCHS/SOL. Participants without diabetes (normoglycemia) were defined as having fasting glucose < 5.6 mmol/L or HbA1c < 38 mmol/mol and aged over 40. If those under 40 years old were glucose < 5.6 mmol/L or HbA1c < 38 mmol/L, we excluded the participants from the analysis. Participants with diabetes were defined based on ADA criteria^11^ (by medication, report diagnosis, fasting glucose ≥ 7 mmol/L or HbA1c ≥ 48 mmol/mol), or random glucose > 11.11 mmol/L, and aged ≥ 25 years at the time of diagnosis (to avoid potential misclassification between T1D and T2D).

**Blood pressure** was measured using a standardized protocol. Participants were considered hypertensive when they met at least one of the following criteria: 1) SBP ≥ 140 mmHg, 2) DBP ≥ 90 mmHg, 3) any antihypertensive medication reported, or 4) ICD-9 codes 401. x or ICD-10 codes I10.x - I15.x. ^12^

**Cardiovascular diseases.** Some of the PAGE participating cohorts have information (prevalence, incidence, or death) on cardiovascular diseases. ARIC, MEC, and WHI ascertained the prevalence or incidence of myocardial infarction (MI) and stroke. Detailed descriptions of CVD ascertainment by studies are reported in the **following section**.

**CVD Ascertainment by PAGE-participating studies**

In ARIC, information on the CHD events including hospitalization and deaths were collected through annual follow-up interviews and community surveillance.^13^ Definitions of CHD events included acute hospitalized MI, definite fatal CHD, MI diagnosed by ECG, and revascularization.^13^

In MEC, As described in previous studies^14^, CHD cases and controls from several nested case-control substudies in MEC will be used in the current proposal. CHD cases were ascertained through the participants’ medical record from the California Hospital Discharge Data (1990 - 2012) and the Centers for Medicare and Medicaid Services claim files (outpatients) (1999 - 2011), which were linked to MEC study - c.f., some participants from Hawaii (76.6% of Japanese American) were not available for hospital discharge data. Case definitions for CHD were ICD-9 codes (DX 410 - 414) for ischemic heart disease as the principal or first diagnosis code and the principal or first procedure code. Also, if a primary cause of death is MI (ICD-9 DX410, ICD-10 I21) or other CHD (ICD-9 DX411-414, ICD-10 I20, I22-25), these individuals were included as cases. Both prevalent (~20%; ascertained at baseline) and incident (~80%; ascertained during follow-up) CHD cases were ascertained.^13^ Controls were selected among those without history of heart attack or angina from the questionnaire at baseline or all follow-up questions.

In WHI, CHD events were identified through self-reported questionnaire and adjudicated by physicians after reviewing the chart within 3 months.^15^ CHD cases were defined as individuals who had a history of MI (self-reported) or a revascularization procedure at baseline, and/or manifested a definitive MI, went through a revascularization procedure, or died from CHD during follow-up. ^15^

**GWAS of BMI and lipid traits in UKBB**

GWAS analysis was conducted using SAIGE^16^ and a linear mixed model – with a kinship matrix as a random effect and covariates as fixed effects. Continuous traits were rank-based inverse normalized within each ancestry group, and covariates in the GWAS included age, sex, age*sex, age^2^, age^2^*sex, and the first 10 PCs (<https://github.com/atgu/ukbb_pan_ancestry/wiki/QC>).

**Performing local genetic correlation analyses using LAVA** **^17^**

Protective and adverse BMI-lipid bivariate loci were identified by local genetic correlation analysis using a pair of UKBB GWAS summary statistics for obesity (BMI) and lipid traits (HDL, LDL, and TG). Local genetic correlation analyses were conducted using the *LAVA* R package. A total of 3 obesity-lipid trait pairs were analyzed.

LAVA, like other local genetic correlation estimation tools, was developed to estimate the locus-level genetic correlation between two phenotypes. The following summarizes the approach described in the original method paper.^17^ LAVA first estimates the local genetic signal (measured by local heritability (h^2^)) as follows.^17^

$$Y_{p}=X\alpha_{p}+\epsilon_{p}$$

$Y_{p}$ : Standardized phenotype vector

X: genotype matrix with $K_{snp} SNPs$ (standardized)

$\alpha_{p}:$ vector of joint SNP effects (accounting for LD)

$\epsilon_{p}$ : vector of normally distributed residuals with variance $\eta_{p}^{2}$

$\hat{\alpha}_{p}=\left( X^{T}X \right)^{-1}X^{T} \boldsymbol{Y}_{\boldsymbol{p}}$ , if the local SNP LD matrix is denoted as $S=cor(X)$ and the vector of estimated marginal SNP effects are denoted as $\hat{\beta}_{p}$ (not accounting for LD), $\hat{\alpha}_{p}=S^{-1}\hat{\beta}_{p}$. That is, if marginal SNP effects are obtained from GWAS summary statistics we can estimate the joint SNP effects ($\hat{\alpha}_{p})$ using a reference population’s LD structure. Using the estimated joint SNP effects, local residual phenotypic variance ($\eta_{p}^{2})$ and the proportion of phenotypic variance explained by the SNPs wihtin the locus (local h^2^) can be estimated. Then, it estimates bivariate local genetic correlations. The local genetic effects (G) can be defined as $G=X\alpha$ ($\alpha$ is a K (number of SNPs in the locus) by P (number of phenotypes) matrix of joint SNP effects). The realized covariance matrix of G is denoted as follows ($\Omega)$.

$$\Omega=\left( \begin{matrix} \omega_{p}^{2} & \omega_{qp} \\ \omega_{pq} & \omega_{p}^{2} \end{matrix} \right)$$

$\omega_{p}^{2}$ : local genetic variance of $G_{p}$ for phenotype p

$\omega_{pq}$: local genetic covariance of $G_{p}$ and $G_{q}$ for phenotype p and q

Then, the local $r_{g}$can be calculated by $\rho_{pq}= \frac{\omega_{pq}}{\sqrt{\omega_{p}^{2}\omega_{q}^{2}}}$, and $\rho_{pq}^{2}$ is considered as the proportion of variance in the local genetic effects $G_{p}$ explained by $G_{q}$. Since G is not actually observed, $\Omega$ should be estimated using the Method of Moments, not computed directly. Significance of the correlation is determined using simulation-based p-values. This local genetic correlation analysis is especially useful for the situations where some signals appear in opposing directions at different regions and nullify each other in a global level – i.e., absensce of global genetic correlation despite the presence of local genetic correlation in opposing directions, whereas global genetic correlation captures only the average genetic correlation across the whole genome and sometimes cannot differentiate the null genetic correlation.^17^

LAVA utilizes pre-partitioned genomic regions to get a local genetic correlation estimate for each locus. We used 2,495 pre-partitioned genomic regions that has been provided by the developers of LAVA (<https://github.com/cadeleeuw/lava-partitioning>). These partitioned genomic blocks were generated based on the 1000 Genome European reference population on build hg19/GRCh37 to get approximately LD-independent genomic blocks across the whole genome.

As described earlier, LAVA first performed the univariate test to filter in the loci where a significant local genetic influence (measured by local heritability (h^2^)) on adiposity or lipid traits was estimated. It excluded the loci without any significant local heritability for either of the two traits from the following bivariate analysis (correlation analysis). Then, local genetic correlation coefficients between a pair of obesity traits and lipid traits were estimated among the significant univariate loci.

We defined the bivariate loci as follows. Bivariate loci were genomic regions showing significant local heritability estimates (Bonferroni-corrected p < 0.00002 (=0.05/2,495); call it as “univariate loci”) and local genetic correlation coefficients (Bonferroni-corrected p < 0.05 / number of tested loci (univariate loci) for each obesity-lipid pair). We classifed the bivariate loci into two different groups based on their directions of association with dyslipidemia risk. In other words, if a given bivariate locus shows positive local genetic correlation coefficients between obesity and dyslipidemia (i.e., r_g_ < 0 for HDL-BMI, r_g_ > 0 for LDL-BMI and TG-BMI pairs), the locus was classified as a adverse BMI-lipid bivariate locus whereas if the locus shows negative local genetic correlation coefficients between obesity and dyslipidemia (i.e., r_g_ > 0 for HDL-BMI, r_g_ < 0 for LDL-BMI and TG-BMI), the locus was classified as a protective BMI-lipid bivariate locus.

**REFERENCE**

1. The ARIC Investigators. The Atherosclerosis Risk in Communities (ARIC) Study: design and objectives. The ARIC investigators. *Am J Epidemiol.* 1989;129(4):687-702.

2. Gottesman O, Kuivaniemi H, Tromp G, et al. The Electronic Medical Records and Genomics (eMERGE) Network: past, present, and future. *Genet Med.* 2013;15(10):761-771.

3. Friedman GD, Cutter GR, Donahue RP, et al. CARDIA: study design, recruitment, and some characteristics of the examined subjects. *J Clin Epidemiol.* 1988;41(11):1105-1116.

4. Lavange LM, Kalsbeek WD, Sorlie PD, et al. Sample design and cohort selection in the Hispanic Community Health Study/Study of Latinos. *Ann Epidemiol.* 2010;20(8):642-649.

5. Kolonel LN, Henderson BE, Hankin JH, et al. A multiethnic cohort in Hawaii and Los Angeles: baseline characteristics. *Am J Epidemiol.* 2000;151(4):346-357.

6. The Women's Health Initiative Study Group. Design of the Women's Health Initiative clinical trial and observational study. The Women's Health Initiative Study Group. *Control Clin Trials.* 1998;19(1):61-109.

7. Fesinmeyer MD, North KE, Ritchie MD, et al. Genetic risk factors for BMI and obesity in an ethnically diverse population: results from the population architecture using genomics and epidemiology (PAGE) study. *Obesity (Silver Spring).* 2013;21(4):835-846.

8. Zubair N, Graff M, Luis Ambite J, et al. Fine-mapping of lipid regions in global populations discovers ethnic-specific signals and refines previously identified lipid loci. *Hum Mol Genet.* 2016;25(24):5500-5512.

9. Hu Y, Graff M, Haessler J, et al. Minority-centric meta-analyses of blood lipid levels identify novel loci in the Population Architecture using Genomics and Epidemiology (PAGE) study. *PLoS Genet.* 2020;16(3):e1008684.

10. Expert Panel on Detection E, Treatment of High Blood Cholesterol in A. Executive Summary of The Third Report of The National Cholesterol Education Program (NCEP) Expert Panel on Detection, Evaluation, And Treatment of High Blood Cholesterol In Adults (Adult Treatment Panel III). *JAMA.* 2001;285(19):2486-2497.

11. American Diabetes Association Professional Practice C. 2. Classification and Diagnosis of Diabetes: Standards of Medical Care in Diabetes-2022. *Diabetes Care.* 2022;45(Suppl 1):S17-S38.

12. Wojcik GL, Graff M, Nishimura KK, et al. Genetic analyses of diverse populations improves discovery for complex traits. *Nature.* 2019;570(7762):514-518.

13. Tcheandjieu C, Zhu X, Hilliard AT, et al. Large-scale genome-wide association study of coronary artery disease in genetically diverse populations. *Nat Med.* 2022;28(8):1679-1692.

14. Ke W, Rand KA, Conti DV, et al. Evaluation of 71 Coronary Artery Disease Risk Variants in a Multiethnic Cohort. *Front Cardiovasc Med.* 2018;5:19.

15. Curb JD, McTiernan A, Heckbert SR, et al. Outcomes ascertainment and adjudication methods in the Women's Health Initiative. *Ann Epidemiol.* 2003;13(9 Suppl):S122-128.

16. Zhou W, Nielsen JB, Fritsche LG, et al. Efficiently controlling for case-control imbalance and sample relatedness in large-scale genetic association studies. *Nat Genet.* 2018;50(9):1335-1341.

17. Werme J, van der Sluis S, Posthuma D, de Leeuw CA. An integrated framework for local genetic correlation analysis. *Nat Genet.* 2022;54(3):274-282.
